# Ageing and pituitary neuroendocrine tumours (PitNETs): from bench to bedside

**DOI:** 10.1530/ERC-25-0535

**Published:** 2026-04-01

**Authors:** Dario De Alcubierre, Francesca Carbonara, Tiziana Feola, Francesca Gianno, Giuseppe Minniti, Vincenzo Esposito, Marie-Lise Jaffrain-Rea

**Affiliations:** ^1^ Neuromed IRCCS, Pozzilli (IS), Italy; ^2^ Department of Experimental Medicine, Sapienza University of Rome (RM), Rome, Italy; ^3^ Department of Biotechnological and Applied Clinical Sciences, University of L’Aquila (AQ), L’Aquila, Italy; ^4^ Department of Radiological, Oncological and Anatomic Pathology, Sapienza University of Rome (RM), Rome, Italy; ^5^ Department of Neurology and Psychiatry, La Sapienza University of Rome, Rome, Italy

**Keywords:** PitNET, pituitary tumour, ageing, elderly, senescence

## Abstract

**Graphical Abstract:**

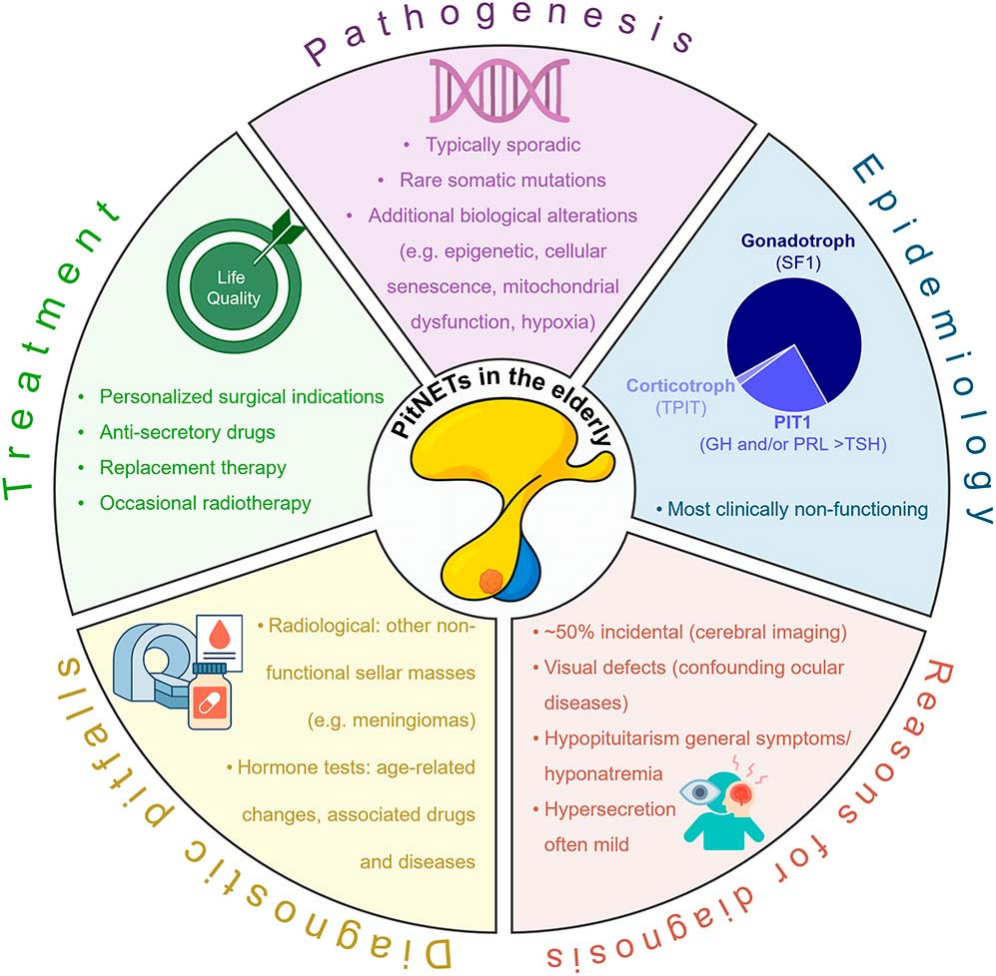

**Abstract:**

The prevalence of pituitary neuroendocrine tumours (PitNETs) in elderly people (≥65 years old) is raising with life expectancy. Despite an increasing incidental detection on neuroimaging for unrelated conditions, a large majority are macrotumours (≥1 cm) and mass effects are frequent, in particular visual defects. Clinically non-functioning PitNETs are the most prevalent (about 75%), and a majority are gonadotrophs. The clinical presentation of functioning PitNETs differ from younger patients and include somatotroph tumours – often intrasellar with mild acromegaly, prolactinomas – often large and invasive, and rare corticotroph and uncommon thyrotroph tumours. Such epidemiological characteristics likely reflect different biological features as compared with PitNETs diagnosed in younger patients. Epigenetic alterations are more frequent than mutations, whereas cell senescence and age-related changes in immune surveillance and feedback mechanisms from ageing target organs may play an underestimated role. Endocrinological changes associated with ageing and concomitant pathological conditions should be considered as relevant confounding factors for diagnosis. In particular, ocular diseases may mask visual defects, progressive symptoms of hypopituitarism may be misinterpreted as ageing, and peripheral physiological (menopause) or pathological dysfunction or drugs given for other diseases can alter pituitary hormone secretion. Mild hypersecretion should be searched for in clinically non-functioning cases, as it may significantly impact on clinical management. Multidisciplinary evaluation is necessary to define personalized therapeutic goals, and where indicated, surgery should be performed by experienced pituitary surgeons. The development of innovative diagnostic and prognostic markers would usefully support the clinical management and follow-up of PitNETs in elderly patients.

## Introduction

Life expectancy has increased significantly over the past century, leading to a growing proportion of older adults in developed countries. Although the definition of ‘elderly’ is not universally standardized, the current WHO definition identifies elderly subjects as aged ≥ 65 years, a population that is expected to increase by 2.9% annually and to reach ∼1.6 billion worldwide by 2050 (https://www.census.gov/en.html). This demographic shift is accompanied by an increasing prevalence of age-related diseases. More than 50% of cancers are currently diagnosed in elderly patients (1). The incidence of pituitary neuroendocrine tumours (PitNETs) also increases with age (2), and the expanding use of imaging modalities results in a higher detection rate, including asymptomatic or minimally symptomatic cases (3). Overall, up to 14% of PitNETs are diagnosed in elderly patients (4), with age-related peculiarities reflecting the predominance of clinically non-functioning (cNF) tumours. In functioning cases, the clinical syndrome of hormone hypersecretion is often mild (e.g. acromegaly) or absent (e.g. post-menopausal prolactinomas). Because signs and symptoms of hypopituitarism often overlap with those associated with ageing, and visual field defects may be masked by common ocular diseases (e.g. cataract and glaucoma), the diagnosis is often delayed, translating into a high proportion of large tumours with visual impairment and/or hypopituitarism (3, 5). The endocrinological evaluation of elderly patients should consider age-related changes in the pituitary gland, due to reduced mass and blood supply, increased connective tissue, hypothalamic changes and/or adaptive responses to the ageing of peripheral target glands (6, 7, 8). Functional alterations include impaired somatotroph function, also known as ‘somatopause’; a reduced sensitivity of corticotroph cells to glucocorticoid feedback; a post-menopausal increase in gonadotropin secretion in women; potential late-onset hypogonadism in men; and changes in the hypothalamic–pituitary–thyroid axis. Diagnostic pitfalls in basal and dynamic tests also result from systemic comorbidities, including metabolic, inflammatory or neurological disorders and their treatment (7). Chronic comorbidities may also increase the anaesthesiologic risk and limit surgical eligibility, although, based on appropriate patient selection and surgical expertise, post-operative outcomes have considerably improved over the past decades (9). On the other hand, the slow-growing pattern of most PitNETs and a satisfactory response to anti-secretory drugs in functioning cases may favour conservative options. Yet, the biology of PitNETs and their potential translational impact in elderly patients have been poorly investigated. This review aims to provide the basis for a personalized multidisciplinary approach and opens new avenues for further research in this field.

### Epidemiology

The distribution of PitNETs in the elderly has been essentially characterized in surgical series reporting the outcomes of transsphenoidal surgery (TS) in elderly or very elderly patients (9, 10, 11, 12), thereby introducing a surgical recruitment bias. The epidemiology of clinically relevant PitNETs in this age group can be partially inferred from population databases (e.g. SEER), including surgical and non-surgical patients (2). However, the distribution and peculiarities of PitNETs in non-surgical series of elderly patients have rarely been reported (13).

**Figure 1:**
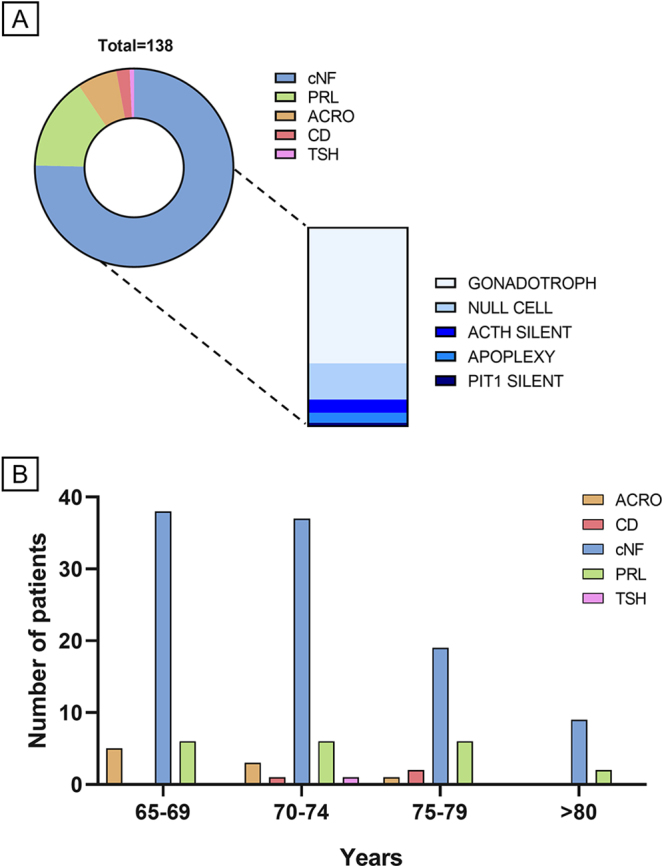
Phenotypic distribution of PitNETs in an Italian cohort of elderly patients. (A) Pie chart illustrating the clinical distribution of PitNETs subtypes in 138 elderly patients and the pathological distribution of 61 cNF PitNETs treated by surgery. Note that ‘null cell’ tumours are overestimated due to some hormone-negative cases untested for pituitary transcription factors. (B) Phenotypic distribution of PitNETs by age at diagnosis. Clinically functioning cases were defined by unequivocal hyperprolactinaemia (PRL) and/or bio-clinical evidence of acromegaly (ACRO), Cushing’s disease (CD) or hyperthyroidism with inappropriate TSH secretion (TSH). PitNETs, pituitary neuroendocrine tumours; cNF, clinically non-functioning.

We therefore elected to illustrate this review by a snapshot of elderly patients (≥65 years at diagnosis) among those followed up for PitNETs at our pituitary unit in 2024 (Neuromed, Italy). We identified 138 patients, with a mean age at diagnosis of 70.0 years (±5.6, SD) and a male predominance (*n* = 83, 60.1%), accounting for 20.8% of this cohort (*n* = 664). The observed phenotypic distribution of PitNETs is shown in Fig. 1. Incidental findings (52.7%) slightly predominated over symptomatic patients coming to our attention because of clinical pituitary dysfunction, headache or visual impairment (47.5%). Only 17 patients had microtumours (12.3%), 10 of which were found incidentally and 7 presented with a pituitary hypersecretion syndrome. A large majority had macrotumours (121/138, 87.6%), including 9 giant PitNETs (≥4 cm). cNF-PitNETs predominated (104/138, 75.3%). Sixty patients (57.7%) underwent TS, and according to pathological reports, a majority were gonadotrophs (*n* = 41, 68.3%), followed by ‘null cell’ tumours (*n* = 11, 18.3%) – which is overestimated due to cases operated on before the routinary implementation of immunostaining for pituitary transcription factors (TFs) – and a minority of silent corticotroph (*n* = 4, 6.6%) or PIT1 (*n* = 1, 1.6%) tumours. Pituitary apoplexy occurred in three patients. Noteworthily, prolactinomas were the second most frequent type in all age groups (21/138, 15.2%) and, although most were macrotumours (18/21, 85.7%), nearly half were found incidentally (10/21, 47.6%). The predominance of male patients (17/21, 80.9%) contrasts with our previous experience, in which 8/9 of elderly prolactinomas were found in women (7). This likely reflects an increased awareness of prolactinomas in peri-menopausal women, with a decrease in inappropriate diagnoses of ‘early menopause’. Somatotroph PitNETs were the third most frequent (*n* = 9, 6.5%), 7/9 revealed by acromegalic changes. Most were intrasellar (7/9, 4 microtumours). Cushing’s disease was rare (*n* = 3, 2.2%) and all caused by macrotumours. One patient was diagnosed with a large and invasive thyrotroph tumour, misdiagnosed for years following thyroidectomy and presenting with iatrogenic hyperthyroidism and inappropriate TSH levels. Brief illustrating case reports will be shown in agreement with medical privacy standards and upon patient consent.

### Biology of PitNETs

The pathogenesis of PitNETs is multifactorial. Despite the rarity of aggressive or metastatic tumours (ag/met PitNETs), invasive and recurrent cases are frequent (14) and several hallmarks of cancer can be recognized (15). Among these, hereditary factors are more prevalent in young patients and poorly considered in the elderly. Nonetheless, one of our patients was affected by a large invasive cNF-PitNET with hypercalcaemic hyperparathyroidism and turned out to carry a familial *MEN1* mutation. Four patients belonged to familial isolated pituitary adenoma (FIPA) kindreds. Thus, despite a relatively low prevalence in the elderly (3.6% in our series), inherited predisposition may be suspected on a clinical basis. Somatic alterations in PitNETs are being increasingly linked to their lineage of origin (16). Except for some ag/met PitNETs, which are uncommon in the elderly, tumour mutational burden is typically low, with no hotspot pathogenic mutation identified in the most prevalent gonadotroph subtype (14). Chromosomal losses or gains predominate in secreting tumours, whereas gonadotroph PitNETs generally exhibit a ‘quiet’ genome (16). Thus, the prevalence of genetic abnormalities appears to be low in most elderly PitNETs. Because ageing and tumourigenesis share common biological features beyond genetic instability, including a time-dependent accumulation of cellular damage, epigenetic alterations, cellular senescence, telomere or mitochondrial dysfunction and hypoxia (17), we briefly reviewed the features of cellular ageing and their potential contribution to the biology of PitNETs in the elderly.

Epigenomic changes are strongly associated with ageing, with a global hypomethylation at CpG islands and hypermethylation in cancer (18). As compared to the normal pituitary, PitNETs generally display a global DNA hypomethylation, with significant variations in the spectrum of demethylated genes according to their lineage of origin (15). Methylome clusters are associated with transcriptional signatures and behavioural features, including tumour size and invasiveness (19). Noteworthily, DNA hypomethylation occurs more frequently in PIT1-PitNETs, particularly in somatotroph tumours (16). Additional epigenetic changes include histone modifications and an altered expression of miRNAs and other non-coding RNAs (20). Several miRNAs targeting key components of cell cycle and senescence signalling are differentially dysregulated in PitNETs (21).

Senescence is induced by stressful events leading to a stable arrest in cell proliferation, with an increase in cyclin kinase inhibitors and a reduced phosphorylation of pRb. DNA damage, increased beta-galactosidase activity and the secretion of several cytokines and chemokines defining the senescence-associated secretory phenotype are reported. Senescence, which is typically more prevalent in pre-malignant lesions but eluded in fully malignant neoplasms, may participate in the common indolent course of PitNETs (14). For example, somatotroph PitNETs are associated with a p53/p21 senescence pathway. An impaired immune clearance may also contribute to the age-related accumulation of senescent cells (22). T-cell senescence is a key feature of immune ageing and contrasts with an increase in other immune cells determining a low-grade chronic inflammation, possibly induced in part by the accumulation of senescent cells (23). Transcriptomic analyses of two PitNETs cohorts also revealed decreased T lymphocyte and increased neutrophil populations in tumours from elderly patients (24).

Cell cycle arrest triggered by telomere dysfunction and activation of the DNA damage response occur as telomeres progressively shorten with the increasing number of cell divisions throughout life. Neoplastic cells frequently escape replicative senescence by maintaining telomere length through enhanced telomerase – mainly because of mutations or epigenetic changes dysregulating the *TERT* gene – or through ALT – which may be associated with ATRX/DAXX defects. If *TERT* alterations seem poorly relevant in PitNETs, ALT and/or loss of ATRX may occur in ag/met cases (25, 26), which are uncommon in the elderly.

Hypoxia also contributes to a functional decline during ageing and acts through the stimulation of p21 by the AMPK signalling pathway, leading to an arrest in the G1/S phase (27). PitNETs are less vascularized compared to the normal pituitary, which may contribute to their slow proliferation rate unless adaptative responses occur (e.g. activation of the HIF-1α pathway and angiogenesis). Whether PitNETs are more hypoxic in the elderly than in younger patients is unclear, but apoplexy may be more frequent, especially in men in their 6th–7th decade (13, 28). Hypoxia may also trigger alternative splicing, which is able to activate oncogenic pathways in tumours with low mutation rates, including PitNETs (29). An overexpression of HIF-1α – which also induces mitochondrial dysfunction – has been associated with a more invasive behaviour in PitNETs (30).

Ageing is linked to a high susceptibility of mitochondria to morphological changes, potentially associated with dysfunction and resulting in oxygen radical damage. The accumulation of mitochondria translates into oncocytic changes, which occur in normal and neoplastic pituitary cells, in particular gonadotrophs (31). Accordingly, the prevalence of ‘oncocytomas’ is higher among gonadotroph PitNETs and increases with age (32).

Such observations encourage further studies to elucidate differential pathogenetic mechanisms of PitNETs in the elderly. The potential role of feedback from ageing peripheral organs, in particular of gonadal dysfunction in the predominance of gonadotroph PitNETs, has not been specifically addressed. The identification of prognostic molecular markers on surgical samples and of innovative diagnostic/therapeutic markers through a liquid biopsy (30) would be of special relevance to achieve a more comprehensive tumour characterisation, allowing personalized decision-making and follow-up of mostly non-functioning tumours.

### Gonadotroph and non-functioning tumours

A progressive age-related increase in cNF-PitNETs occurs in adults of both sexes, achieving nearly 80% of PitNETs in the elderly (4, 7). A large majority of operated cases are classified as gonadotrophs (GnPitNETs) by immunostaining for FSH and/or LH, additional cases being diagnosed by nuclear SF1 and/or GATA3 expression (33). Functioning GnPitNETs are rare and yet not reported in the elderly, which can be explained by ageing gonads, especially in women. Silent corticotroph PitNETs, the second most common form of cNF-PitNETs in the elderly, will be discussed later on. Noteworthily, many studies reporting the characteristics and prognosis of cNF-PitNETs suffer from heterogeneous clinical or pathological definitions. Subclinical hypersecretion may be missed by basal hormone evaluation, and the systematic use of pituitary TFs in hormone-negative PitNETs has dramatically reduced the proportion of ‘null cell’ tumours (1–2%) (33). Further prospective studies would therefore be useful to re-evaluate the prognostic value of silent secreting and ‘null cell’ subtypes. From a clinical point of view, cNF-PitNETs generally present an indolent course, many elderly patients appear asymptomatic at the time of diagnosis, which is increasingly made incidentally on cerebral imaging for cognitive impairment or falls, or during the workup of malignancies (4). Nonetheless, mass-related effects are often present but under-evaluated or misattributed to other age-related conditions, and visual field defects and hypopituitarism are more prevalent and severe in the elderly (5, 7, 11, 12). The slow rate of tumour progression may contribute to the age-related delay in the etiological diagnosis of visual symptoms (34). Nonetheless, the size of cNF-PitNETs in the elderly is generally similar if not smaller (35) than in younger patients, with lower rates of parasellar invasion (4, 11, 12).

TS remains the primary treatment for symptomatic cNF-PitNETs in the elderly, even in their 9th decade (10). In the past decade, tumour resection rates were reported to be comparable to non-elderly patients (4, 5, 11, 12, 36). However, age-related considerations should be made: i) the main objective is to relieve compression symptoms and debulking may be preferred to total resection depending on the context, ii) a large series suggest a higher incidence of post-operative complications compared to younger patients, including cerebrospinal fluid leakage, visual worsening or cranial nerve palsies (4), cranial complications (e.g. post-operative haematoma) (36), post-operative electrolyte imbalance and delayed hyponatraemia – possibly favoured by drugs for unrelated conditions (11), and iii) an age-related increase in perioperative mortality due to circulatory, respiratory and infectious causes has also been observed (5, 11, 36, 37). An unexpectedly high age-related increased risk of mortality was reported in a single study, due to transcranial and/or non-elective surgery (37). Nonetheless, based on appropriate patient selection and surgical expertise, TS is generally safe and the choice between active surveillance and surgery should be tailored on an individual basis, taking into account chronic comorbidities and anaesthesiologic risk, life expectancy and mass effects. Patients should be informed about expected benefits and risks of surgery, including a lower chance to improve pre-operative pituitary dysfunction as compared to younger patients translating into a potential long-life replacement therapy for corticotroph and/or thyrotroph deficits – the higher risk of developing new deficits remains controversial (11, 34). The risk of post-operative AVP deficiency is not clearly influenced by age (11). Visual recovery rates may be comparable to non-elderly patients (70–100%) (5, 11, 12, 35), although lower recovery rates have also been reported, possibly reflecting a longer duration of optic nerve compression (4, 35). Thus, high-volume referral centres with multidisciplinary management are recommended to optimize surgical outcome (4, 5, 11, 12, 36).

**Figure 2:**
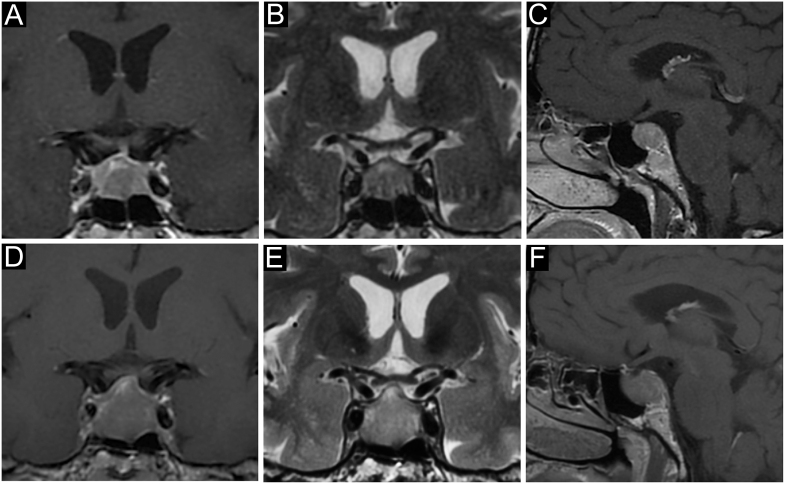
A case of non-functioning gonadotroph PitNET in a 65-year-old man. This patent was referred because of an incidental sellar mass on cerebral MRI for unexplained episodes of brief unconsciousness, in the setting of recent hypertension. Pituitary MRI was consistent with a non-invasive PitNET with initial suprasellar extension (A, B, C)*. Clinical examination, endocrinological evaluation and visual field (VF) examination were unremarkable, and follow-up was advised. A slow worsening of pituitary function was noticed over the next 4 years, requiring L-thyroxine and then transdermal testosterone replacement. A slow progression towards the optic chiasm was noticed (D, E, F)*, and transsphenoidal surgery was planned despite normal VF. Noteworthily, minimal temporal quadrantopsia was noticed just before surgery. No recurrence was found at a 2-year follow-up, and transdermal testosterone was recently withdrawn upon urological advice because of a worsening benign prostate hypertrophy. *(A and D) coronal contrast-enhanced T1-weighted, (B and E) coronal T2-weighted and (C and F) sagittal contrast-enhanced T1-weighted.

An important – but often uncertain – clue in the management of cNF-PitNETs in the elderly is their evolutive potential. In clinical practice, a frequent issue is to encourage – or not – an elderly patient referred for an asymptomatic macrotumour abutting the optic chiasm to plan TS before some new comorbidity appears and increases the operative risk in case of tumour progression and/or symptoms. Compared with younger patients, a slower growing rate has been consistently reported in the elderly (38, 39), translating into a longer progression-free survival (40), a significantly lower recurrence rate at 5 years (7% vs 15.5%) (40, 41), a lower rate of re-operation (3.8 vs 9.5%) or adjuvant radiation therapy (12), although these latter points might also be attributable to comorbidities and prioritization of quality of life (4). However, as illustrated in Fig. 2, the need for careful follow-up to detect tumour progression should be acknowledged by patients and their entourage. Of note, pathological subtyping matters since silent corticotroph PitNETs are frequently invasive and carry a high of recurrence (42).

### Prolactinomas

Almost all lactotroph PitNETs are functioning and commonly designed as prolactinomas. They are poorly represented in surgical series and predominate in males in the elderly (43). Their true prevalence may be underestimated due to first-line medical treatment, as suggested by our cohort (nearly 15%). They are functionally asymptomatic in post-menopausal women because of ovarian failure and sexual symptoms are generally under-reported or misattributed to ageing in men. Diagnosis delay may therefore contribute to their characteristics at diagnosis, with mass effects revealing large, often invasive tumours. About 5% are revealed by pituitary apoplexy, and intra-tumoural heterogeneity is frequently observed at MRI due to necrosis or haemorrhage (44, 45). Extension to the skull base may suggest a parasellar tumour, in particular a chordoma, pointing out the importance of pre-operative PRL determination, considering serum dilution (e.g. 1:100) to avoid a potential ‘hook effect’. Nonetheless, they are increasingly found incidentally (43). Noteworthily, unlike premenopausal prolactinomas, most of which are microtumours and improve or regress after menopause (46), elderly prolactinomas in women are typically macrotumours, one-third reporting a previous history of secondary amenorrhoea (7, 44, 45). Because untreated microprolactinomas rarely evolve to macroprolactinomas, this suggests some differential biological characteristics in post-menopausal tumours.

Distinct biological features may act together with a common diagnostic delay to explain the large size of elderly prolactinomas at diagnosis. Lactotroph PitNETs in men are usually larger and more invasive than in women at any age, with molecular/chromosomal abnormalities associated with aggressive features (47). Male and post-menopausal female lactotroph PitNETs share a reduced expression of oestrogen receptor alpha (ERα), which may reflect a lower degree of cell differentiation and have a negative prognostic impact (47). Thus, sex–gender differences seem to be attenuated in the elderly.

**Figure 3:**
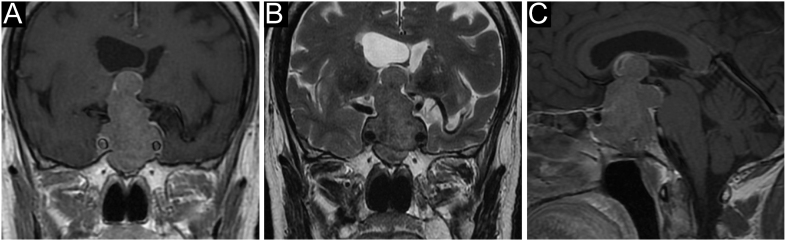
A giant invasive lactotroph PitNET in a 77-year-old man. This patient presented with fatigue, worsening headache and visual impairment, in the absence of significant medical history. Bitemporal hemianopsia was found at visual field examination, and brain MRI revealed a giant and invasive pituitary mass (maximal diameter 62 mm). Endocrine evaluation showed very high plasma PRL levels (20,000 ng/mL after dilution 1:100) with hypopituitarism. The patient was started on glucocorticoid and thyroxine replacement therapy with a low starting dose of cabergoline (CAB, 0.25 mg/week) and gradual titration up to 2 mg/week according to PRL values. A rapid clinical and visual response was observed, with a progressive reduction in tumour volume. PRL normalization was achieved within 1 year of treatment. The tumour went on shrinking to a small, intrasellar and cystic remnant (7 mm), and CAB was progressively reduced to a maintenance dose (1 mg/wk). The bio-clinical and radiological features remained stable over an 8-year follow-up. (A) Coronal contrast-enhanced T1-weighted scan, (B) coronal T2-weighted scan and (C) sagittal contrast-enhanced T1-weighted scan.

Dopamine agonists (DAs), in particular cabergoline (CAB), represent the first-line treatment for macroprolactinomas (43). Male prolactinomas are more frequently resistant to standard pharmacotherapy and more likely to require a multimodal approach (46). However, despite frequent long-term dependence on treatment, DAs are very effective in elderly patients, with PRL normalization and tumour shrinkage achieved in up to 83% of cases (43). In our cohort, except a single case of first-line TS because of severe mass effects, prolactinomas were successfully managed with first-line long-term CAB treatment, including a giant tumour (Fig. 3). Unlike pre-menopausal prolactinomas in women, in whom DA discontinuation is recommended based on the very low risk of tumour regrowth (<5%), post-menopausal prolactinomas deserve prolonged DA treatment to mitigate the risk of tumour enlargement (46). It is commonly considered that hyperprolactinaemia is no longer a concern after menopause. However, indirect benefits of treating hyperprolactinaemia on bone density, body composition and metabolism would argue for maintaining PRL in the normal range (46). PRL is also mitogenic for the breast. Because no consensus is reached about optimal PRL values in the elderly, we propose to taper the treatment to the minimal effective weekly (or bimonthly) dose before considering withdrawal and maintain PRL within the normal range through annual PRL assays, which also limits indications for neuroradiological follow-up.

### Acromegaly

Despite peaking in the third/fourth decade of life, an age-related increase in the incidence of acromegaly reaching 14.8–18.2 per 100.000 inhabitants among people over 65 years old has been reported (48). The Liège acromegaly survey (LAS) showed a significant increase in the age of diagnosis over time and an equal gender distribution (49). Elderly acromegalics generally present with a mild disease, with some clinical signs and symptoms overlapping with ageing features, lower basal and post-glucose nadir GH, lower age-corrected IGF1 and less frequent PRL co-secretion (7, 48, 50, 51). Despite diagnostic delay (52), mass-related symptoms are less common than in younger patients due to smaller, less invasive, often intrasellar tumours (49, 51, 53, 54).

Conversely, systemic complications are more prevalent in elderly acromegalics, due to overlapping age-related conditions, and may significantly impact on therapeutic choices. Not surprisingly, an age-related impairment in glucose metabolism has been reported, with a higher proportion of diabetes mellitus in most series (48, 49). This likely reflects the progressive inability of ageing beta-cells to cope with disease-related insulin resistance. Cardiovascular diseases are also more common, with a significantly higher prevalence of hypertension and left ventricle hypertrophy compared to younger patients and age-matched controls and a higher prevalence of systolic and diastolic dysfunction (50). The prevalence and severity of obstructive sleep apnoea (OSA) are also higher, possibly due to additional effects of GH/IGF1 hypersecretion on the weakening of the upper airway musculature (52). Age was also identified as an independent risk factor for cancer in acromegaly (55). Conversely, the impact of age on the acromegalic osteoarthropathy and disease-related vertebral fractures is less documented (52, 55), with an age-related risk of any fracture reported only in women (49).

**Figure 4:**
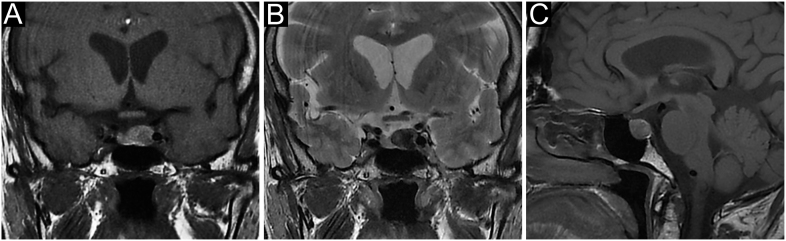
An intrasellar somatotroph PitNET in a 68-year-old woman. This patient presented with a 3-year history of increasing headache and coarse features, acral enlargement, prognathism and paraesthesia. Elevated plasma GH (5.9 ng/mL) and IGF1 (510 ng/mL, 1.8 × ULN) confirmed acromegaly with normal PRL levels (7.7 ng/mL). A 15 mm intrasellar, non-invasive pituitary tumour was found at MRI, hypointense on T2-weighted scans. She had a long-standing thyroid goitre, hypertension and impaired fasting glucose, a recent diagnosis of carpal tunnel syndrome and a recent unexplored breast mass, which was immediately referred for biopsy, revealing a ductal carcinoma. Thus, she received first-line somatostatin receptor ligands treatment to allow surgery and radiotherapy for breast cancer, and GH/IGF1 was already controlled when she underwent transsphenoidal surgery. Pathological examination concluded for a GH/PRL-secreting PitNET. After post-operative remission, cabergoline was recently introduced (1.5 mg/week) for slightly elevated IGF1 (1.2 ULN) and anti-aromatase treatment is ongoing with no evidence of oncological recurrence. (A) Coronal contrast-enhanced T1-weighted scan, (B) coronal T2-weighted scan and (C) sagittal contrast-enhanced T1-weighted scan.

TS, which may offer a chance of cure and improvement of systemic complications, remains the first-line treatment in elderly acromegalics (48). No increase in peri-operative complications and comparable, if not higher, post-operative remission rates have been reported as compared to younger patients, owing to smaller tumour size (53, 54). Nonetheless, cardiorespiratory comorbidities may lead to consider first-line medical treatment with somatostatin receptor ligands (SRLs), which may also lower the anaesthesiologic risk (48). Other comorbidities, such as cancer (Fig. 4), may also limit or delay surgery. Several studies have reported a higher sensitivity of elderly acromegalics to SRLs compared to younger patients, with a rate of disease control reaching 50–55% (48, 52), possibly due to a higher proportion of densely granulated tumours and increased SSTR2 expression (54). This is particularly relevant if patients are not candidates for surgery. In our series, patients received either first-line TS or long-term first-generation SRLs with satisfactory disease control. Experience with the second-generation SRL pasireotide and the GH receptor antagonist pegvisomant – alone or in combination with first-generation SRLs – is still limited in the elderly, but appears safe and effective (52). However, the risk of pasireotide-associated hyperglycaemia could be higher, making pegvisomant a more attractive option due to its beneficial effects on glucose control (56). The IGF1 response to pegvisomant may not be influenced by age (57). Despite the lack of specific evidence in the elderly, disease control in acromegaly has been associated with an increased life expectancy (48) and a shift in mortality causes, more similar to the general population (55).

### Corticotroph PitNETs and Cushing’s disease

Cushing’s disease (CD), the most common form of endogenous hypercortisolism, is prevalently diagnosed in the third/fourth decade with a 4:1 female predominance. With ageing, CD becomes less frequent than other aetiologies of Cushing’s syndrome and the female-to-male ratio decreases near to 1:1 (58). In the elderly, CD is rare and typically presents with subtler symptoms and fewer signs of overt hypercortisolism. A retrospective study of baseline features in 608 CD patients revealed a significant age-related decline in the aggregate number of hallmark CD features (≤2 vs 2.5–3.5 in younger patients) (59). In particular, weight gain, typical facial rounding, *striae rubrae* and dorsal–cervical fat pad were significantly less prevalent, consistent with previous observations of lower weight gain, BMI and waist circumference in older individuals (58). A more pronounced catabolic phenotype is also suggested by greater muscle wasting, exacerbating age-related frailty and impaired physical functions (58, 60). The reduced prevalence of clinical hyperandrogenism (i.e. acne, hirsutism and hair loss) may also reflect an age-related decline in adrenal androgen secretion (59). Conversely, older patients with CD tend to exhibit more comorbidities, with a higher prevalence of hypertension, diabetes mellitus, bone fractures, venous thromboembolism, cardiovascular diseases (58, 60), dyslipidaemia, OSA and infections (59). The prevalence of comorbidities has been estimated to increase 1.1- to 4.7-fold compared to the normal ageing population (59), confirming the role of hypercortisolism in the development of cardiometabolic diseases (61).

**Figure 5:**
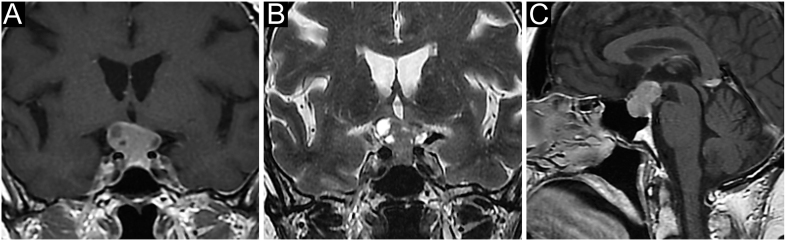
An invasive ACTH-secreting macrotumour in a 76-year-old woman. This patient was referred for the endocrinological evaluation of a sellar/suprasellar mass, incidentally revealed by cerebral imaging after a domestic fall complicated by epistaxis. The patient complained of unexplored weight gain (7–8 kg over the past 18 months) and increased pharmacological requirement for the treatment of hypertension. Mild Cushingoid features were observed, with facial rounding, central obesity, a BMI of 34.2 kg/m^2^ and peripheral muscle wasting. ACTH-dependent hypercortisolism was confirmed by elevated urinary free cortisol levels (1.7 × ULN), lack of suppression of serum cortisol following overnight 1 mg dexamethasone (7.5 μg/dL) and elevated ACTH concentration (142.7 pg/mL). A pituitary macrotumour with suprasellar extension and cystic changes was found at pituitary MRI (A, B, C). Transsphenoidal surgery was performed and pathological examination confirmed diffuse ACTH immunoreactivity with low Ki-67 (1%) in the absence of p53 expression. Post-operative headache occurred due to a modest suprasellar haematoma, which progressively regressed. Bio-clinical post-operative remission of hypercortisolism occurred despite a small tumour remnant. Three years later, a mild bio-clinical recurrence of hypercortisolism occurred with tumour regrowth. The patient received stereotactic radiotherapy and cabergoline (CAB, up to 2 mg/week) with tumour stabilization and bio-clinical normalization of hypercortisolism. Three years later, escape to CAB led to introduction of low-dose osilodrostat (up to 1 mg × 2 daily) with a good tolerance and control of hypercortisolism. However, very last MRI follow-up is suggestive for disease progression, which is under multidisciplinary re-evaluation. (A) Coronal contrast-enhanced T1-weighted scan, (B) coronal T2-weighted scan and (C) sagittal contrast-enhanced T1-weighted scan.

The biochemical diagnosis of CD may be complicated by several age-related factors, including impaired glucocorticoid feedback and biorhythm with an age-related increase in late-night salivary cortisol (62). Increased hypothalamus–pituitary–adrenal axis activity is also frequently associated with dementia or depression, and renal failure may impair cortisol clearance. Several medications (*e.g*. statins and antidepressants) alter CYP3A4 activity and complicate the interpretation of cortisol suppression tests due to impaired dexamethasone metabolism (58). The 1.8 μg/dL cut-off for normal cortisol suppression after 1 mg dexamethasone overnight may therefore be too restrictive in elderly subjects (7). As compared to younger patients, elderly patients with CD may exhibit higher late-night salivary cortisol concentrations (62) but lower ACTH relative to cortisol (63). The 24 h urinary free cortisol measurement is also less likely to support CD diagnosis (87% vs 95% in younger cohorts), often falling within the normal range due to reduced filtration rates and/or a more indolent disease (58, 59). Nonetheless, older CD patients have been more frequently reported with macrotumours, suggesting age-related differences in tumour biology and/or delayed diagnosis (Fig. 5). A continuum likely exists between clinically functional and silent corticotroph tumours, which represent the second most frequent type of cNF-PitNETs in this age group (34). The positive association between age and pituitary tumour volume or Knosp grade (58, 63), contrasting with a decrease in Ki-67 (59), would support a generally slow-growing potential of these tumours.

Although TS remains the treatment of choice for CD, offering a chance of cure, older patients are more likely to be managed conservatively and less frequently by first-line TS (68.9% vs 89.3% in younger patients) (58, 60). The outcome of TS has been variably appreciated, with remission rates reported to be lower (58), comparable (60) or even better (64) than in younger patients. In the presence of mild pre-operative hormone hypersecretion, radiological evidence of residual disease in the absence of clear post-operative hypercortisolism may confound the definition of surgical remission, as illustrated in Fig. 5, pointing out the role of MRI during follow-up. Noteworthily, with the exception of deep vein thrombosis (59), post-operative complications appear to be similar to younger patients (60, 63, 65). Thus, appropriate patient selection upon multidisciplinary evaluation and systematic anti-thrombotic prevention is especially relevant for neurosurgical safety in elderly CD patients. Conservative treatment mainly relies on pharmacological treatment and, in selected cases, radiotherapy (RT). Among adrenal steroidogenesis inhibitors, ketoconazole has demonstrated comparable efficacy and safety in patients aged over 75 years (66). Despite very limited data reported on metyrapone and osilodrostat, the latter is being increasingly used (63). Pituitary-directed agents, pasireotide and CAB, appear safe and effective in older patients (67), but glucose metabolism and valvular disease should be contemplated. Because the efficacy of RT on the control of hormone hypersecretion has been variably appreciated, all other second-line options should be considered (68).

### Thyrotroph PitNETs

**Figure 6:**
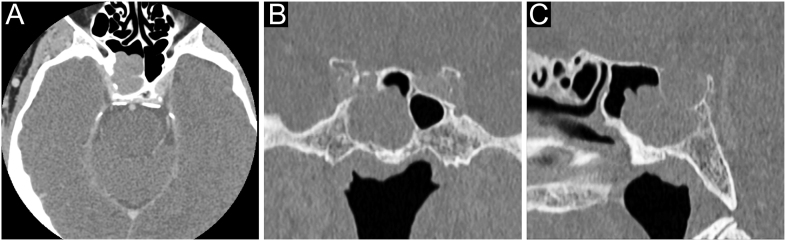
A TSH-secreting PitNET in a 74-year-old man. This patient came to our observation with a previous history of goitre and hyperthyroidism treated with anti-thyroid medications followed by total thyroidectomy. He had received increasing doses of levothyroxine for persistently elevated TSH and had a pacemaker implanted for the management of arrhythmias and atrial fibrillation. Due to iatrogenic hyperthyroidism, replacement therapy was reduced from 175 to 75 μg/dL, leading to a further increase in plasma TSH (15 µIU/mL) when peripheral euthyroidism was restored. Basal PRL and GH were normal (17 and 1.3 ng/mL, respectively) with low IGF1 levels (45 ng/mL). He denied headaches or visual disturbances, but a contrast-enhanced head CT revealed a sellar mass (27 × 21 mm) with parasellar and infrasellar invasion. Transsphenoidal surgery was then performed, and pathological findings concluded for a mixed TSH/PRL immunoreactivity with no mitoses and Ki-67 expression (3%). Based on post-operative residual biochemical and radiological disease, the patient was successfully treated with first-generation somatostatin receptor ligands, with a good tolerance and efficacy over a 4-year follow-up. (A) Coronal contrast-enhanced T1-weighted scan, (B) coronal T2-weighted scan and (C) sagittal contrast-enhanced T1-weighted scan.

TSH-secreting PitNETs are rare (<2%) and predominantly diagnosed around the 5th decade but may occur at any age without gender predominance (69, 70). Clinical manifestations typically include hyperthyroidism, goitre, more rarely atrial fibrillation and heart failure, and/or mass effects due to the high prevalence of macrotumours (69, 70). Inappropriate TSH secretion is characterized by normal or slightly increased plasma TSH concentrations (3–12 μU/mL in a review of 535 cases) with elevated free thyroid hormones levels (70), which should be distinguished from inherited resistance to thyroid hormones (69). In patients who had previous thyroidectomy for recurrent hyperthyroidism (Fig. 6), goitre or thyroid carcinoma, elevated FT4 levels due to administration of replacement therapy before sampling should also be excluded (69, 70). Guidelines from the European Thyroid Association recommend first-line surgery, provided that euthyroidism is restored pre-operatively by anti-thyroid drugs and/or SRLs (69), which is of special importance in elderly patients due to the added burden of thyrotoxicosis on cardiovascular risks. Because first-generation SRLs restore euthyroidism in >90% and induce significant tumour shrinkage in up to 50% of the patients (69), they may represent a suitable first-line option for inoperable tumours (71). Complete surgical resection may be hampered by tumour invasiveness or fibrosis, requiring long-term SRL treatment and RT in selected patients (70).

### Aggressive and metastatic PitNETs

Ag/met PitNETs can occur at any age but are rare in the elderly. Indeed, most are diagnosed in the 5th decade of life, with a large majority of lactotroph and corticotroph tumours and an under-representation of gonadotroph tumours (72). In our series, despite some challenging/recurring cases, no tumour fulfilled the current clinical definition of the ag/met PitNET, with the recent potential exception of a corticotroph tumour shown in Fig. 5. Interestingly, an 83-year-old female patient received long-term temozolomide for an aggressive corticotroph PitNET (47 cycles) with an excellent clinical response in the absence of major side effects (73).

### Special therapeutic considerations in elderly PitNETs

As discussed hitherto, a major issue in elderly patients is to make a balanced decision about surgery based on patient’s age, physical conditions and expectations. Overall, the outcome may be comparable to those of younger patients (74), with significant symptom relief and low mortality rates (65) despite a slightly higher risk of post-operative complications (3). If surgery is elected to improve life quality or expectancy, high-volume centres offering experienced neurosurgeons and multidisciplinary expertise are recommended to optimize surgical results while minimising intra-/post-operative risks. Regular follow-up and collaboration with general practitioners or geriatrists are also essential to optimise patient management.

The indications and goals of replacement therapy for hypopituitarism differ from younger patients (7). Briefly, adrenal replacement therapy is mandatory in the presence of corticotroph insufficiency, avoiding overtreatment because of potential adverse effects on blood pressure, glucose metabolism or bone health, but providing appropriate education of the patient and entourage on the prevention of acute adrenal insufficiency. The treatment of overt hypothyroidism (based on low FT4 concentrations) should also avoid over-treatment to prevent cardiac arrhythmias – especially atrial fibrillation – and deleterious effects on bone metabolism. Based on recommendations for the treatment of hypothyroidism in the elderly and in the absence of reliable TSH monitoring, it appears reasonable to maintain FT4 in the low-normal range. Gonadal replacement therapy may be safely considered in men once prostate cancer, recent vascular events and frailty are excluded, and upon close prostate and haematocrit monitoring (75). Transdermal testosterone should be preferred to intramuscular formulations, checking plasma testosterone after transdermal application to ensure appropriate replacement avoiding supraphysiological peaks. Low-dose GH replacement therapy (GH-RT) may also be considered in the elderly based on potential pleiotropic beneficial effects on health and a favourable impact on body composition and quality of life (76). Because elderly patients are especially sensitive to GH and more prone to develop side effects than younger adults, and very limited data are available in patients aged ≥ 80 years, the cost-to-benefit ratio of GH-RT should be carefully evaluated – and periodically re-assessed – on an individual basis (76). As daily GH-RT may also be a concern in patients receiving multiple medications, long-acting formulations may offer an advantage, which should be more specifically addressed in the elderly (77). Finally, attention should be paid in patients with AVP deficiency to impaired thirst and use of concomitant drugs increasing the risk of dehydration or electrolyte imbalance (e.g. diuretics and SGLT2 inhibitors).

The role of RT in elderly patients with PitNETs remains poorly investigated. Evidence from other benign intracranial tumours suggests that medium-term adverse effects are comparable to those observed in younger patients (78), while elderly individuals may be less exposed to long-term complications, such as secondary malignancies and cerebrovascular complications. Modern RT techniques include stereotactic radiosurgery (SRS), typically delivered in a single fraction of 13–25 Gy, and conventionally fractionated stereotactic radiotherapy (FSRT), usually administered at total doses of 45–54 Gy in 25–30 daily fractions of 1.8–2.0 Gy. As in younger patients, the choice is primarily based on tumour size, invasiveness and proximity to the optic pathways. Although cognitive outcomes after pituitary irradiation remain poorly characterized in older adults, hippocampal-sparing strategies should be considered when feasible (79). Patients should also be followed up for clinically relevant delayed post-radiation hypopituitarism.

Both SRS and FSRT achieve long-term tumour growth control in approximately 75–90% of PitNETs at 5 and 10 years, respectively (7). In cNF-PitNETs, RT is generally indicated after TS in cases of progressive residual disease or recurrence, aiming to offer long-term tumour control and avoiding repeat surgery. In functioning PitNETs, the biochemical remission of hormonal hypersecretion following RT is less predictable, ranging from 40 to 75% in different series (7). In acromegaly, biochemical remission often requires several years and is influenced by baseline GH and IGF1 levels, with higher levels making remission harder, especially in larger tumours; consequently, the prolonged time to hormonal normalization may limit the clinical benefit of RT in elderly patients (7). Conversely, hormonal control may be achieved more rapidly in corticotroph PitNETs, albeit at the cost of higher recurrence rates (Castinetti *et al.* (68)). In prolactinomas, RT is rarely indicated due to the high efficacy and good tolerability of DA and reserved to selected medically resistant tumours. Therefore, pharmacological therapy with anti-secretory agents should be fully optimized before considering RT in functioning PitNETs, including thyrotroph tumours (70).

## Conclusion

PitNETs are being increasingly found in elderly patients, who represent a heterogeneous population including a subset of very elderly and/or frail patients with relevant comorbidities. The diagnosis may be fortuitous or delayed due to confounding symptoms and concomitant drugs, or conditions may impact on the evaluation of pituitary function. An appropriate diagnosis of hormone hypersecretion, which is often mild, is essential because most functioning PitNETs in the elderly may benefit from surgical resection and/or appropriate anti-secretory drugs. Because a large majority are non-functioning, the therapeutic decision and follow-up will largely depend on tumour extension and repeated imaging, considering patient’s age, health and life expectancy. Where deemed useful and feasible, TS can be offered safely in pituitary reference centres with multidisciplinary support. A better understanding of the molecular biology of PitNETs in the elderly would help guide patient management and prognostic stratification. Besides hormone evaluation in functioning tumours, new tissue and circulating biomarkers will hopefully help define the effective need for surgery in indolent cases and define an appropriate frequency of imaging for both operated and non-operated cases.

## Declaration of interest

The authors declare that there is no conflict of interest that could be perceived as prejudicing the impartiality of the work reported.

## Funding

This work was partially supported by Current Research from Neuromed IRCCS (Pozzilli, IS) and Project ECS 0000024 Rome Technopole, CUP B83C22002820006, PNRR Missione 4 Componente 2 Investimento 1.5, funded by NextGenerationEU to TF.

## Author contribution statement

MLJR and DDA conceived and designed the study. DDA, MLJR and FC wrote the manuscript. DDA, FC and FG performed illustrations. TF, FG and VE revised and commented on the manuscript. MLJR supervised the study. All authors approved the final version of the manuscript.
